# DIGITAL STORYTELLING AND INTERGENERATIONAL COLLABORATIONS: OLDER ADULTS AND COLLEGE STUDENTS

**DOI:** 10.1093/geroni/igac059.977

**Published:** 2022-12-20

**Authors:** Angela Lavery

**Affiliations:** West Chester University, West Chester, Pennsylvania, United States

## Abstract

The use of digital storytelling can be a helpful tool within community work, health and social work research and policy. Digital storytelling refers to life-story telling that can be done in a variety of ways and used to encourage social change and transformation. This presentation will include experience on how this method was used in a study and an intergenerational project between older adults and university graduate and undergraduate students. This group of older adults specifically shared their experiences with equine interactions and activities, while the university students worked with the older adults to create a digital story. For this study and project, recruitment included students enrolled in different disciplines. Discussion on digital storytelling’s connection to the narrative method and critical gerontology framework will be noted. Challenges and barriers, including Institutional Review Board and ethical considerations while preparing for this method will also be discussed.

